# Book Reviews

**Published:** 1901-01

**Authors:** 


### BOOK REVIEWS, PAMPHLETS, EXCHANGES.

### BOOK REVIEWS.

[Contributions solicited for review.]

SEXUAL DEBILITY IN MAN. By Frederick R. Sturgis, M.D., Formerly Clinical Professor of Venereal Diseases, Medical Department, University of the City of New York; Ex-Visiting Surgeon to the City Hospital, Blackwell's Island; Author of "A Manual of Venereal Diseases"; one of the Authors of "A System of Legal Medicine," etc., etc. E. B. Treat & Co., Publishers, 241-243 W. 23d St., New York. Price, $3.00 net.

Dr. Sturgis, whose name is well known as that of one of the foremost workers in genito-urinary diseases, has written a very readable and helpful book. His style is original and attractive, and displays erudition and experience. The volume strongly appeals to one for its honesty of expression and its method of setting forth the facts and theories of the subject. We can heartily commend it, and advise any one who is interested in this branch of medicine to become the possessor of the book. It will be of undoubted aid to him, and by its general attractiveness will invite a deeper interest in the subject.

EGBERT'S HYGIENE AND SANITATION. A Manual of Hygiene and Sanitation. By Seneca Egbert, A.M., M.D., Professor of Hygiene in the Medico-Chirurgical College of Philadelphia. New (2d) and Revised Edition. In One Handsome 12mo Volume of 427 pages, with 77 Engravings. Cloth, $2.25 net. Lea Brothers & Co., Publishers, Philadelphia and New York.

This volume offers a comprehensive outline of this very important subject. As the physician is in such large measure the custodian of public health, he should be well informed on sanitary matters. This book is a good one from which to gain the necessary information. The subject is considered under the following heads: Bacteriology, atmosphere, ventilation and heating, water, food, stimulants and beverages, personal hygiene, school hygiene, disinfection and quarantine, removal and disposal of sewage, military hygiene, vital statistics, examination of air, water and food.

---

MEDICAL DISEASES OF INFANCY AND CHILDHOOD. By Dawson Williams, M.D. (Lond.), Fellow of the Royal College of Physicians of London, and of University College, London, etc. Second Edition, Revised, with Additions by Frank Spooner Churchill, M.D., Instructor in Diseases of Children in Rush Medical College in Affiliation with the University of Chicago; Professor of Pediatrics in the Chicago Polyclinic. Illustrated with 72 Engravings and two Colored Plates. Lea Brothers & Company, Philadelphia and New York, 1900.

This work will be found very helpful in pediatric practice. It is clear and practical, and contains much that is valuable as a result of the author's experience. Artificial feeding of infants and disorders of digestion are fully considered. The American addition has been edited to include the views entertained by American practitioners.

---

A TEXT-BOOK OF THE PRACTICE OF MEDICINE. By James M. Anders, M.D., Ph.D., LL.D., Professor of the Practice of Medicine and of Clinical Medicine, Medico-Chirurgical College, Philadelphia, etc. Fourth edition. Octavo, pp. 1292. Illustrated. Philadelphia and London: W. B. Saunders & Co.

The rapid appearance of the fourth edition attests the popularity of Anders' Practice. It has been adopted as a text-book in a number of institutions and well deserves the distinction. The fourth edition represents a revision and amplification of the former issues. Additions have been made of the chapter on Diseases of the Digestive Organs, and those on Ileocolitis in Children and Acute Cholecystitis have been rewritten.

---

A TEXT-BOOK OF HISTOLOGY, including Microscopic Technic. By A. A. Boehm and M. von Davidoff, Munich. Edited with additions by G. Carl Huber, University of Michigan. Translated from the Second German Edition by H. H. Cushing, Philadelphia. W. B. Saunders & Co., Philadelphia. Price, $3.50.

This book is marked by thoroughness and technical excellence. It is profusely illustrated, containing not only the original illustrations, but many more added by the American editor. Those parts of the work concerned with the glandular and nervous system are particularly full. The laboratory technic is eminently commendable. The book will be especially valuable to students and histological workers, by whom its merits will be readily appreciated.

---

A TEXT-BOOK UPON THE PATHOGENIC BACTERIA. For Students and Physicians. By Joseph McFarland, M.D., Professor of Pathology in the Medico-Chirurgical College, Philadelphia, etc. With 142 Illustrations. Third Edition, Revised and Enlarged. Philadelphia: W. B. Saunders & Company, 1900. Price, $3.25 net.

The selection of the proper text-book on this subject is not easy in view of the number from which to choose. McFarland's Bacteriology has enjoyed great popularity, and as it contains the elements necessary to a work of this character, the student and laboratory worker would consult his interest by choosing it. It clear and comprehensive, full and complete in all important essentials. There is no other work in the field that better fulfills its purpose.

---

THE MEDICAL NEWS VISITING LIST FOR 1901. Weekly (dated, for 30 patients); Monthly (undated, for 120 patients per month); Perpetual (undated, for 30 patients weekly per year); and Perpetual (undated, for 60 patients weekly per year). The first three styles contain 32 pages of data and 160 pages of blanks. The 60-patient Perpetual consists of 256 pages of blanks. Each style in one wallet-shaped book, with pocket, pencil and rubber. Seal Grain Leather, $1.25. Thumb-letter Index, 25 cents extra. Philadelphia and New York: Lea Brothers & Co.

This handsome visiting list is known to all practitioners. The present issue meets all that is required of an indispensable adjunct to practice.

---

THE PHYSICIAN'S VISITING LIST (Lindsay & Blakiston) for 1901, now ready and for sale by all booksellers and druggists. P. Blakiston's Son & Co., Philadelphia. Price from $1 to $2.25.

This publication has just reached its fiftieth year, a fact of which its publishers may well feel proud. It is certainly one of the best, if not the best of its kind.

PROGRESSIVE MEDICINE, VOL. III., September, 1900. A Quarterly Digest of Advances, Discoveries and Improvements in the Medical and Surgical Sciences. Edited by Hobart Amory Hare, M.D., Professor of Therapeutics and Materia Medica in Jefferson Medical College of Philadelphia. Octavo, handsomely bound in cloth, 408 pages, with 14 engravings. Lea Brothers & Co., Philadelphia and New York. Issued quarterly. Price, $10 per year.

The progress of medicine is well set forth in this volume. The articles are thoroughly practical and instructive. The contributors to Vol. III. are William Ewart, M.D., F.R.C.P., on Diseases of the Thorax and Viscera; W. G. Spiller, M.D., Diseases of the Nervous System; Henry W. Stelwagon, M.D., on Diseases of the Skin; Richard C. Norris, M.D., on Obstetrics.

---

CLINICAL EXAMINATION OF THE URINE AND URINARY DIAGNOSIS. A Clinical Guide for the Use of Practitioners and Students of Medicine and Surgery. By J. Bergen Ogden, M.D., Instructor in Chemistry, Harvard University Medical School, etc. Illustrated. Pages 416. Price, $3 net. W. B Saunders & Co., Philadelphia. 1900.

This book is intended as a clinical guide for diagnosis through the urine. It is divided into two parts: one treating of the normal and abnormal constituents of the urine; the other of diagnosis. It is one of the best works on this very important subject and deserves popularity.

---

BACTERIOLOGY AND SURGICAL TECHNIQUE FOR NURSES. By Emily M. A. Stoney, Superintendent of the Training School for Nurses, St. Anthony's Hospital, Rock Island, Ill. Philadelphia: W. B. Saunders & Co. 1900.

The book is well printed, indexed and illustrated and contains all that is required in the way of instruction to nurses, especially those who are concerned in surgical work. The work can be commended to them.

